# Tuberculosis amidst COVID-19 pandemic in India: unspoken challenges and the way forward

**DOI:** 10.1186/s41182-021-00377-1

**Published:** 2021-10-21

**Authors:** Mainak Bardhan, Mohammad Mehedi Hasan, Ishita Ray, Anusua Sarkar, Priyanka Chahal, Sudhan Rackimuthu, Mohammad Yasir Essar

**Affiliations:** 1 Division of Bacteriology, ICMR-National Institute of Cholera and Enteric Diseases, Kolkata, India; 2 Department of Biochemistry and Molecular Biology, Faculty of Life Science, Mawlana Bhashani Science and Technology University, Tangail, Bangladesh; 3 Mahatma Gandhi Memorial Medical College, Indore, India; 4 Department of Biotechnology, Heritage Institute of Technology, Kolkata, India; 5 S. Tentishev Asian Medical Institute, Kant, Kyrgyzstan; 6 Father Muller Medical College, Mangalore, Karnataka India; 7 Kabul University of Medical Sciences, 1001 Kabul, Afghanistan

**Keywords:** Tuberculosis, COVID-19, India, Public health

## Abstract

India is home to the most significant number of tuberculosis (TB) cases around the globe. The COVID-19 crisis has deeply perturbed most of the essential TB services in India. Regulating TB is difficult in a densely populated country like India due to latent TB infection in millions of Indians, which can reactivate at any point in the future. Due to the ongoing pandemic, healthcare workers have been diverted to activities implemented for effective COVID-19 management, leaving a meager workforce to help deal with TB management. Integrating TB and COVID-19 to augment India’s health outreach is the need of the hour to diminish the effect of the COVID-19 crisis on TB. Increasing overall testing capacity, active screening, implementation of strategies for easy identification of TB hotspots, and ensuring uninterrupted drug supply for treatment through heedful planning of local and regional distribution and transportation will especially help cater to the vulnerable population who are at a high risk of suffering from adverse outcomes of TB. Lessons learnt in the battle against COVID-19 can most definitely help in providing insights to fulfill the goal of eliminating TB from India.

## Introduction

Tuberculosis (TB) has consistently been a subject of major concern worldwide, and is mostly believed to affect the destitute section of the population [1]. However, immunocompromised individuals like human immunodeficiency virus (HIV) patients, patients on interleukin 17 inhibitor and tumor necrosis factor inhibitor therapy for psoriasis, psoriatic arthritis, ankylosing spondylitis and inflammatory bowel disease are at a significant risk for reactivation of TB regardless of their socioeconomic class [2, 3]. Since TB in India gravely affects young adults in an economically productive age group, it consequently has massive social and economic repercussions [4]. Despite the advent of the latest diagnostic approaches and treatment facilities, TB is still one of the most deadly infectious diseases [5]. The pathogenesis of TB occurs with the transmission of an acid-fast bacilli bacterium called *Mycobacterium tuberculosis* via aerosols or droplets when an inflicted person sneezes, coughs or speaks [1, 6]. After getting infected with TB, an individual might not exhibit any signs or symptoms of the disease immediately. However, there is still a 5–15% chance of developing the active symptomatic illness within 2–5 years from acquiring the pathogen. TB mainly affects immunodeficient patients such as those inflicted with HIV, diabetes mellitus, malnutrition, and SARS-CoV-2, among many others [1, 7]. The symptoms are predominantly cough along with fever, chills, exhaustion, appetite loss, and weight loss. It has been speculated that if TB goes untreated, the potential carrier can infect a further 10–15 people annually [1, 8].

According to the reports in India, TB inflicted 2.64 million people in the year 2019 that had led to the demise of approximately 450,000 people [9]. This indicates that there had been close to 1000 reported deaths each day. India has faced the highest cases of TB in the world, with the number of people affected being 10 million, and the death toll has correspondingly risen to a staggering 1.4 million every year [10]. Nevertheless, this scenario was before the COVID 19 pandemic had hit India. The cases of TB have continuously surged in India owing to its late diagnosis and inadequate infrastructure. The advent of COVID-19 has further caused impediments in timely diagnosis of TB similar to other infectious diseases in the country [9, 11, 12]. As per the available data, in March 2020, the number of children receiving the Bacillus Calmette–Guérin (BCG) vaccine was 260,000 less as compared to the previous year. These values had further gone down in April. The curve had shown slight improvement in May, and by June 2020, 23,000 fewer patients had received the full dose of BCG.

TB and COVID-19 are infectious diseases that share many similarities in their pathogenesis. Their primary organ of attack are the lungs [13]. Apart from it, they also share similar symptomatology like cough, fever, and breathlessness, which can lead to misdiagnosis of either disease. However, this can be differentiated from TB, as TB has a longer incubation period and a more prolonged course than COVID-19. One infection increases the chances of contracting the other due to a weakened host immune system [14]. Nevertheless, when a person suffers from both diseases at the same time, it further complicates the situation. It is advised that while combating COVID-19, the patients should not interrupt the ongoing TB treatment.

## Current efforts and challenges in the context of TB during COVID-19 in India

India has the largest number of TB patients in the world and was recently plagued by the second wave of COVID-19 with utmost severity [14–16]. The COVID-19 crisis has deeply perturbed most of the essential TB services in India. In India, where two deaths occur every 3 min from TB [8], continuing TB services in parallel is vital [17]. In 2018, India had the highest TB incidence (2.7 million) and fatality (0.4 million) globally [18].

Regulating TB is difficult in a densely populated country like India due to latent TB infection in millions of Indians, which can reactivate at any point in the future [19]. Being a developing nation, India is home to a large number of impoverished individuals who are susceptible to developing severe TB infections such as miliary TB. The BCG vaccine, though available to most Indians, is still not sufficient in controlling TB infections and its spread. The protection granted by BCG wanes off 10–20 years after childhood BCG immunization. This fading TB immunity leaves one susceptible to TB in adulthood when the maximum risk of exposure exists [20]. Multidrug-resistant (MDR) and extensive drug-resistant (XDR) TB are other hurdles to overcome TB in India [14, 17]. Other risk factors for TB include air pollution, smoking, inadequate treatment of infection, HIV, overcrowded living conditions, and an increased burden of diabetes that can predispose to severe multi-organ TB infection [19].

The National Tuberculosis Elimination Programme (NTEP) is the main government organization dealing with TB elimination in India. The National Strategic Plan (NSP) for TB elimination 2017–2025 is a framework which guides the national and state governments, civil society organizations, development partners, international agencies, research institutions, private sector, etc., for eliminating TB from India. The new NSP was developed by the NTEP and World Health Organization (WHO) during the Joint Monitoring Mission conducted in 2019. The NSP 2017–2025 which takes lessons from the last NSP, and puts forward novel aggressive steps required to move India towards TB elimination [21].

The strategic pillars of the NSP are ‘Detect–Treat–Prevent–Build’ [10]. The pandemic has disrupted all these four primary components of the NSP.

### Detection

It constitutes finding all drug-sensitive and drug-resistant TB cases in urban and rural areas focusing on undiagnosed TB in high-risk populations [19]. The nationwide lockdown has drastically reduced patient footfall in most government and private sectors, making TB case detection difficult. Moreover, patients with early TB symptoms may not visit health facilities due to fear of contracting SARS-CoV-2. This is a double-edged sword as untreated or inadequately treated TB can make individuals more susceptible to life-threatening COVID-19 infection in the future [17]. The health care workers have been diverted to activities implemented for effective COVID-19 management, leaving a meager workforce to help deal with TB management. Government laboratories and imaging centers are burdened with COVID-19 testing, leaving little to no room for TB diagnostic tests to be conducted. Sample collection is also an arduous task in the era of social distancing. The private sector in India, which may have additional resources to help in TB control, is currently not streamlined. Transportation of lab supplies, samples from patients, and TB drugs have also been disrupted due to the COVID-19 lockdown. This is especially detrimental to TB detection and treatment in remote areas.

### Treatment of TB

For effective treatment, timely initiation of anti-TB drugs and follow-up of all positive patients on the prescribed treatment is necessary. COVID-19 has additionally made it more challenging to locate TB cases. Poor compliance with TB chemotherapy is a significant reason behind the increase in MDR TB and XDR TB incidence in India, and the numbers are expected to be even higher during and post COVID-19 [22]. The WHO had come up with the novel Directly Observed Treatment Short-course (DOTS) strategy to prevent loss to follow-up of TB patients. It involves universal daily regimens for TB cases and scaling up short-course treatment for drug-sensitive and drug-resistant TB. COVID-19 has reduced the availability of DOTS providers and anti-TB drugs. Doctors and government officials supervising TB treatment have been burdened and involved in managing COVID-19 in the country [15], leading to substandard quality of TB programs and activities which are already in place. Lack of prior planning to ensure continuous drug supply to TB patients under emergency conditions led to the system coming to a standstill [14]. Provisions for delivery of anti-tuberculosis treatment drugs to the doorstep of TB patients were announced by the government, however its effectiveness is yet to be ascertained [14, 23].

### Prevent and build

Supervision of TB control in India has been suboptimal, to begin with, due to a paper-based system of recording and reporting [19]. The COVID-19 pandemic has made this process even more tedious. Policy building, high-level political commitment to anti-TB strategies, TB research, developing highly trained human resources, and better surveillance were some of the other targets of NSP for 2017–2025.

### Modifications in NTEP during COVID-19 pandemic

Social distancing strategies during COVID-19 have not only helped curb SARS-CoV-2, but also helped limit TB spread, the aerosols of which remain suspended in the air for 10 days [14]. Bidirectional screening of patients has been recommended by the Ministry of Health and Family Welfare in India, which expedited the diagnosis of both pulmonary TB and COVID-19 [14, 23]. Bidirectional TB–COVID screening is defined as conducting COVID-19 screening for all TB cases and TB screening for all COVID-19-positive cases according to recent guidelines by the Ministry of Health and Family Welfare (MoHFW) [23]. Moreover, both COVID-19 and TB testing is recommended for cases of severe acute respiratory infection and Influenza like illness [23]. TB patients post the advent of COVID were provided anti-tuberculosis treatment drugs for a longer duration to limit multiple visits to DOTS centers. Telemedicine also helps resolving minor TB patient complaints without physically meeting the health care provider [14, 24].

The advent of contact tracing application has helped in combating TB in the wake of COVID-19 [25]. The introduction of COVID-19 tracking mobile application named Aarogya setu by the government of India has sidelined the TB-tracking applications [26]. During the COVID-19 pandemic situation TB should not be neglected. Providing TB medications in this current scenario has been an ordeal; some of the reasons are the shortage of medical supplies and re-stocking of medicines. Multiple approaches to overcome this situation have been engaged, for instance providing medicines to patients by postal services [27]. The WHO has issued a strategy to combat multidrug-resistant TB. This strategy suggests the lessening of tuberculosis prevention administration to daily 1-month administration of rifapentine and isoniazid for those who have been in proximity with TB patients [28]. Recently, the Government of India has recommended the administration of TB drugs for 1 month and in some special cases for 2 months. This step has been enforced to limit the visits to clinics, thereby reducing the chance of transmission [29]. However, all such plans have come to a halt due to the COVID-19 pandemic.

## Recommendations

India aims to end TB by the Year 2025; however, the COVID-19 pandemic is adversely affecting the goal both directly and indirectly. To diminish the effect of the COVID-19 crisis on TB, consistent practices are required to highlight the TB burden amidst the COVID-19 pandemic while taking urgent steps such as (1) Supporting and organizing innovative national and state-level TB control programs; (2) minimizing hospital visits to prevent nosocomial infections, including COVID-19, by promoting house visits and follow-ups to ensure medication compliance; (3) a possible implementation of smartphone technology for quick, easy assessment and monitoring as deemed necessary.

Integrating TB and COVID-19 to augment India’s health outreach is also recommended by collaborating with national STOP TB programs [30]. Increasing overall testing capacity, active screening, implementation of strategies for easy identification of TB hotspots and ensuring uninterrupted drug supply for treatment through heedful planning of local and regional distribution and transportation will especially help cater to the vulnerable population who are at a high risk of suffering from adverse outcomes of TB. Steps can also be undertaken to incorporate or modify the new advanced technologies developed as a result to manage COVID-19 with TB effectively.

It is pertinent to note that despite the difficulties faced as a result of the pandemic, targeted health approaches, structured social policies, safe mental health, and health equity of TB patients as well as others have to be taken into account.

## Conclusion

India being home to the largest number of TB cases around the globe, the burden of the disease must not be undermined even while facing the COVID-19 pandemic. Prevention, testing, tracing, and management strategies of TB patients need to be further restructured and re-evaluated in light of the current state of affairs to adequately meet the needs of TB patients during the ongoing pandemic. Lessons learnt in the battle against COVID-19 can most definitely help in providing insights as to how the country can better the pre-existing programs and strategies to achieve the goal of eliminating TB in India by 2025.

## Data Availability

Not applicable.

## Abbreviations

COVID-19: Coronavirus disease 2019
SARS-CoV-2: Severe acute respiratory syndrome coronavirus 2
TB: Tuberculosis
HIV: Human immunodeficiency virus
BCG: Bacillus Calmette–Guérin
MDR: Multidrug-resistant
XDR: Extensive drug-resistant
NTEP: National Tuberculosis Elimination Programme
NSP: National Strategic Plan
DOTS: Directly Observed Treatment Short-course
WHO: World Health Organization
